# Resistance towards metronidazole in *Blastocystis* sp.: A pathogenic consequence

**DOI:** 10.1371/journal.pone.0212542

**Published:** 2019-02-22

**Authors:** Arutchelvan Rajamanikam, Ho Shiaw Hooi, Madhav Kudva, Chandramathi Samudi, Suresh Kumar

**Affiliations:** 1 Depatment of Parasitology, Faculty of Medicine, University of Malaya, Kuala Lumpur, Malaysia; 2 Department of Medicine, Faculty of Medicine, University of Malaya, Kuala Lumpur, Malaysia; 3 Gastroenterology and Hepatology Specialist Clinic, Pantai Hospital, Kuala Lumpur, Malaysia; 4 Department of Medical Microbiology, Faculty of Medicine, University of Malaya, Kuala Lumpur, Malaysia; California State University Fullerton, UNITED STATES

## Abstract

*Blastocsytis* sp. is a protozoan parasite that has been linked to common gastrointestinal illnesses. Metronidazole, the first line therapy, was reported to show frequent inefficacy. Previously, *Blastocystis* sp. isolated from different population showed varying metronidazole resistance. However, the effect of metronidazole treatment on pathogenic potentials of *Blastocystis* sp. isolated from different populations, which is known to have different gut environment, is unclear. This study investigates the *in vitro* effect of metronidazole on the pathogenic potentials of *Blastocystis* sp. isolated from urban and orang asli individuals. *Blastocystis* sp. ST 3 isolated from symptomatic and asymptomatic individuals were treated with a range of metronidazole concentration. The parasites’ growth characteristics, apoptotic rate, specific protease activity and the ability to proliferate cancer cells were analyzed upon treatment with 0.001 mg/l metronidazole. The study demonstrates that *Blastocystis* sp. isolates showed increase in the parasite numbers especially the amoebic forms (only in urban isolates) after treating with metronidazole at the concentration of 0.001 mg/ml. High number of cells in post-treated isolates coincided with increase of apoptosis. There was a significant increase in cysteine protease of *Blastocystis* sp. isolates upon treatment despite the initial predominance of serine protease in asymptomatic isolates. Metronidazole resistant *Blastocystis* sp. also showed significant increase in cancer cell proliferation. Resistance to metronidazole did not show significant different influence on the pathogenicity between *Blastocystis* sp. isolated from urban and orang asli individual. However, an increase in parasite numbers, higher amoebic forms, cysteine protease and ability to proliferate cancer cells implicates a pathogenic role. The study provides evidence for the first time, the effect of metronidazole towards enhancing pathogenic potentials in *Blastocystis* sp. when isolated from different gut environment. This necessitates the need for reassessment of metronidazole treatment modalities.

## Introduction

*Blastocystis* sp. is a protozoan parasite with a worldwide distribution where more than a billion individuals are estimated to harbor this organism[1]. High prevalence has been reported in developing countries than the urbanized ones[2]. The detection of *Blastocystis* sp. in fecal material has been commonly associated to non-specific gastrointestinal symptoms such as diarrhea, flatulence, abdominal cramps[3] as well as iron deficient anemia [4] and urticarial [5]. However, the pathogenicity of *Blastocystis* sp. remains controversial.

To date, up to 17 subtypes (ST) have been isolated where ST 1–9 are found in human infections. ST3 have been shown to have higher prevalence followed by ST1 and this ST has been commonly incriminated to possess pathogenic potentials. Previous studies investigating on subtype diversity reported that ST 3 is predominantly isolated from patients with gastrointestinal symptoms such as IBS [6] and the solubilized antigens from ST3 was reported to trigger increased proliferation in colon cancer cells[7]. Sporadic studies on pathogenic potential on other STs, namely ST2 and ST4 have surfaced but at a lower consistency [8, 9].

Some studies observed persons infected with *Blastocystis* sp. has higher prevalence but remained asymptomatic and suggested that this parasite could be a member of a healthy gut with long-term colonization [10]. However, other studies demonstrated therapeutic improvement upon clearance of this parasite, which suggest that this parasite has a pathogenic potential and requires treatment [11]. Treatment with metronidazole is reportedly the first-line therapy for eradication of the parasite.

Successful eradication of *Blastocsytis* sp. was seen in many studies [3]. However, reports have also witnessed persistence in symptoms upon treatment and it could be due to the resistance conferred by this organism or the failure of the drug to exhibit complete clearance of the parasite. Various *in vitro* studies have shown evidence of resistance when treated with metronidazole[12]. A previous study have reported an increase in mitochondrion-like-organelle and the formation of amoebic forms when treated with low concentration of metronidazole[13]. *Blastocystis* sp. has also been isolated from different population showed variability in resisting metronidazole[14]. However, there has been no study mounted to assess if treatment has the same effect on the same subtype but isolated from different population groups

Urban and rural populations have showed different gut microflora, which has been attributed to mainly lifestyle factors [15]. A rural individuals’ gut possess greater microbial and functional gene diversity. Urban population however have been reported to have lost this diversity presumably due to diverse and varying lifestyles [16]. A study recently have pointed out that the interaction of gut microbiome with *Blastocystis* sp. could be one of the important factors in metronidazole treatment failure [17]. In the present study, *Blastocystis* sp. isolated from urban population and orang asli population was compared in terms of treatment response and pathogenic potentials to implicate the influence of gut environment in *Blastocystis* sp. treatment.

This study, for the first time extends the investigation to study the effect of metronidazole on phenotypic properties and variation in pathogenic potentials of *Blastocystis* sp. ST 3 isolated from 2 distinct population namely urban and orang asli population. The isolates obtained from each population group were further classified as symptomatic and asymptomatic based on clinical presentation.

## Material and methods

### Source of *Blastocystis* sp.

*Blastocystis* sp. was isolated from urban and orang asli (indigenous) population. Urban samples were obtained from Pantai Hospital, Kuala Lumpur and University Malaya Medical Centre (UMMC), Kuala Lumpur. Urban individuals were approached with a questionnaire to validate that they were truly urban settlers. Orang asli samples were obtained from 3 different villages at the outskirts of Selangor, Malaysia. Samples were collected from the village dwellers directly at their houses in their respective villages. These villagers worked as farmers deeper inside the jungle. Those villagers who are working in nearby towns and return only during the evenings and weekends were excluded from this study. Ethical approval for collection of stool samples was obtained from the Medical Ethics Committee of University Malaya Medical Centre, Kuala Lumpur, Malaysia (20154–1281) according to the Declaration of Helsinki. Collection of samples from orang asli settlements was approved by JAKOA (Department of Orang Asli Development). A written consent was obtained from all the participants prior to sample collection.

Urban individual visiting a doctor with frequent and non-specific gastrointestinal (GI) symptoms (flatulence, diarrhea abdominal cramps and etc.) were regarded as symptomatic and those individuals without any incessant and debilitating GI symptoms were regarded asymptomatic. Whereas for orang asli individuals who experienced any frequent GI symptoms (GI symptoms >3 times a week) were regarded as symptomatic and healthy individuals without any GI symptoms were grouped as asymptomatic. These individuals when questioned said they had not taken any anti-protozoal or anti-diarrheal medication 1 month prior to sample collection. Fecal samples collected from both symptomatic and asymptomatic individuals were screened for parasitic infection using formal ether concentration technique. *Blastocystis* sp. was detected by the *in vitro* culture method using Jones medium as described previously[18]. Only samples with *Blastocystis* sp. as the sole infectious agent was used in this study.

### Culturing metronidazole resistant strain

Day 3 culture of *Blastocystis* sp. ST 3 isolated from urban and orang asli individuals were used for this analysis. The parasite count was standardized to a concentration of 1 x 10^5^ cells/ml in fresh Jones’ medium supplemented with 10% horse serum and range of metronidazole concentration (0.0001, 0.001, 0.01, 0.1, 1 mg/ml). The parasites were observed daily until it reached a growth peak. Parasites, which showed maximum growth, were transferred to 3 ml Jones’ medium containing the same concentration of horse serum along with metronidazole. This was maintained throughout the study. Subsequently, a growth profile was carried out comparing the cells pre- and post-treatment. Cells were then washed and counted to 1 x 10^5^/ml. The cells were counted daily for 10 days. Generation time pre- and post-treatment was calculated for 24 hours as described in previous study [19].

### Apoptotic characterization

Apoptotic characterization was done according to the protocol established previously[20]. Parasites from day 3 cultures were inoculated at a concentration of 1 x 10^5^ cells/ml in 1 ml medium. Cells were counted using tryphan blue exclusion assay to determine the viability. The number of apoptotic, necrotic and viable cells was counted using Apoptosis, Necrotic and Healthy Cell Quantification Kit (Biotium Inc., Hayward, CA, USA) at 4 time points. Cells were harvested and washed twice with 1ml of sterile PBS (pH 7.4) before staining. The manufacturer’s protocol was followed and stained cells were observed under an Olympus BX 51 epifluorescence microscope (Olympus, Wetzlar, Germany) using image analyzer software. The control experiment was day 3 *Blastocystis* sp. culture with PBS replacing the treatment.

### Preparation of solubilized antigen and quantification of protease activity

Antigens were extracted from 3-day-old culture supplemented with metronidazole as outlined in the past study[21]. Protease activity was determined using 1.5mg/ml azocasein solution. The antigens were standardized to 0.1mg/ml. Protease activity was quantified using Azocasein Colorimetric Assay as described previously[22]. A control experiment of only bacterial solubilized antigen was carried out.

### Inhibition assay to determine specific protease activity

Four types of protease, namely, cysteine, serine, aspartic and metalloprotease are the common mixture of parasitic protease. An array of protease inhibitor was deployed to detect the dominant type of protease. Protease inhibitors used were E-64 (Cysteine protease inhibitor), Phenylmethanesulfonyl fluoride (PMSF) (Serine protease inhibitor), Pepstatin A (Aspartic protease inhibitor) and EDTA (metalloprotease inhibitor) (Sigma-Aldrich). In this study, specific protease inhibitors were optimized and used to determine predominant protease. A protocol used in the quantification of protease was applied with supplementation of protease inhibitors at a particular concentration as reported by previous studies [23, 24].

### Colon cell culture and proliferation studies

HCT 116 colonic cancer cells were maintained in RPMI medium supplemented with L-Glutamine, antibiotics and fetal bovine serum (FBS). Cells were standardized to 1000 cells in 100 ml and allowed to grow for 24 hours at 37°C, 5% CO_2_. Next, antigens from *Blastocystis* sp. belonging to urban and orang asli individuals as well as from treated and control parasites were introduced to a final concentration of 0.01 μg/ml and allowed for further incubation. Cell proliferation was analyzed after 48 hours through MTT assay as described previously [7].

### Statistical analysis

All statistical analysis was done through IBM SPSS Statistics Version 21. Student’s t-test was performed to compare the means of each group. Spearman’s correlation test was carried out on viable cells and apoptotic forms. P<0.05 was regarded significant in this study.

## Results

**Fig 1:**
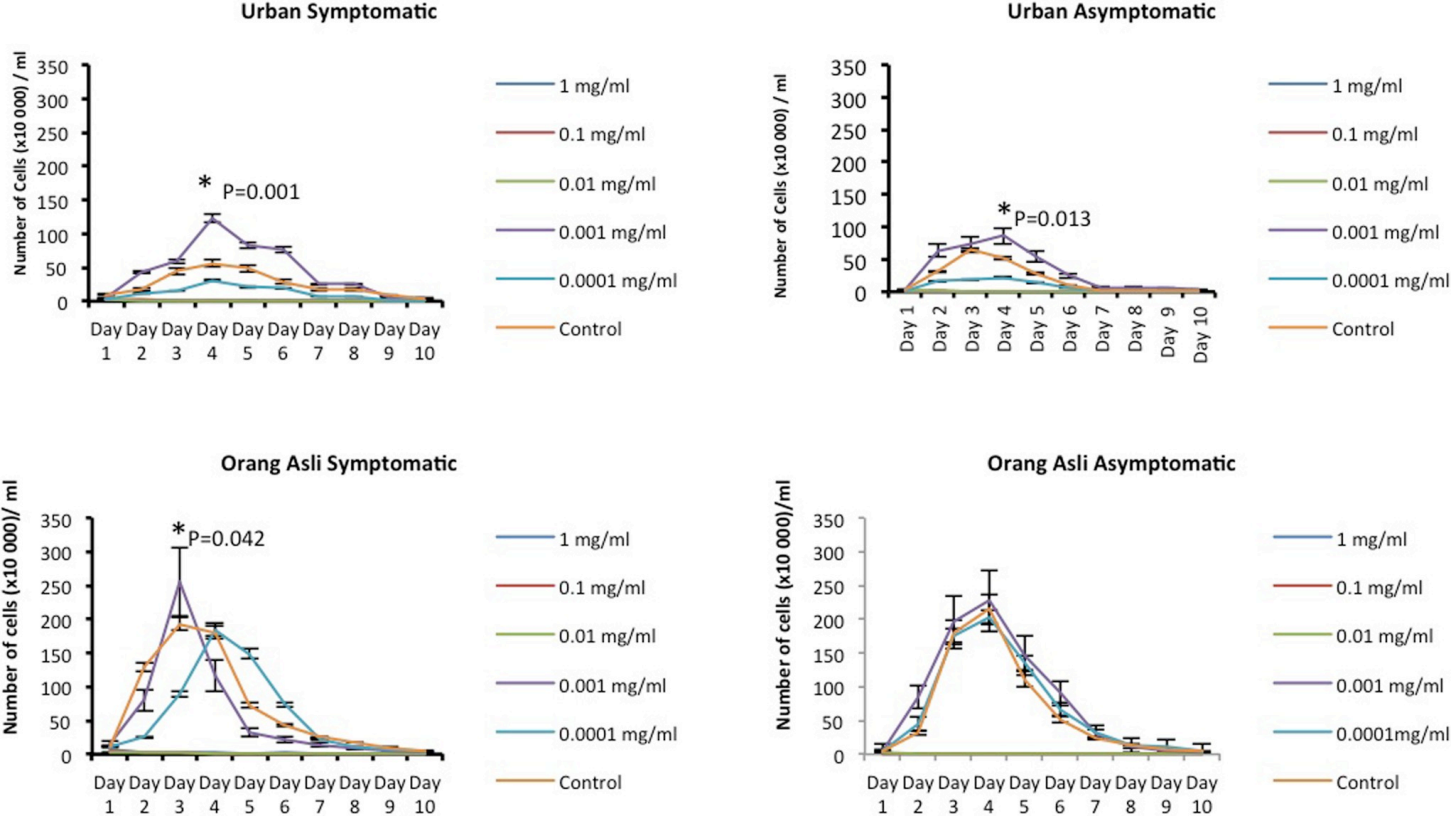
Treatment of *Blastocystis* sp. with various concentration of metronidazole. **Growth profile of symptomatic and asymptomatic isolates from urban and orang asli showed increased proliferation of cells at 0.001 mg/ml of metronidazole**. Values are expressed as mean ± SD of 5 isolates. Cells were counted in 1 ml. Control experiment was done using PBS to replace metronidazole. *P<0.05 in Students t-test for comparison of means of peak cell count between different metronidazole concentrations.

**Fig 2:**
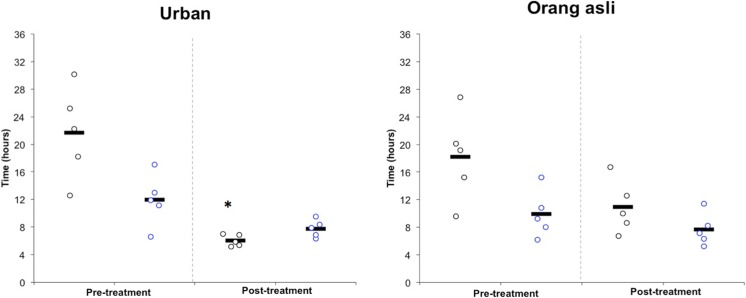
Generation time of *Blastocystis* sp. for control (Pre-treatment) and treated with 0.001 mg/ml metronidazole (Post-treatment). Symptomatic and asymptomatic isolates and represented by black and blue circles respectively. Isolates obtained from symptomatic urban individual showed significant decrease in generation time. The black lines represents mean of 5 isolates. *P = 0.005 for comparisons of mean between control and metronidazole treated *Blastocystis* sp. isolated from symptomatic urban individual.

A range of metronidazole concentrations (1, 0.1, 0.01, 0.001 and 0.0001 mg/ml) was used and cells treated with metronidazole at 0.001 mg/ml was found to show consistent resistance. There was an increase in cell number and decrease of generation time per 24 hour in isolates obtained from both urban and orang asli individuals upon treatment with 0.001mg/ml metronidazole. *Blastocystis* sp. isolated from symptomatic urban and orang asli individuals showed greater increase in cell number compared to asymptomatic isolates (Figs 1 and 2).

**Fig 3:**
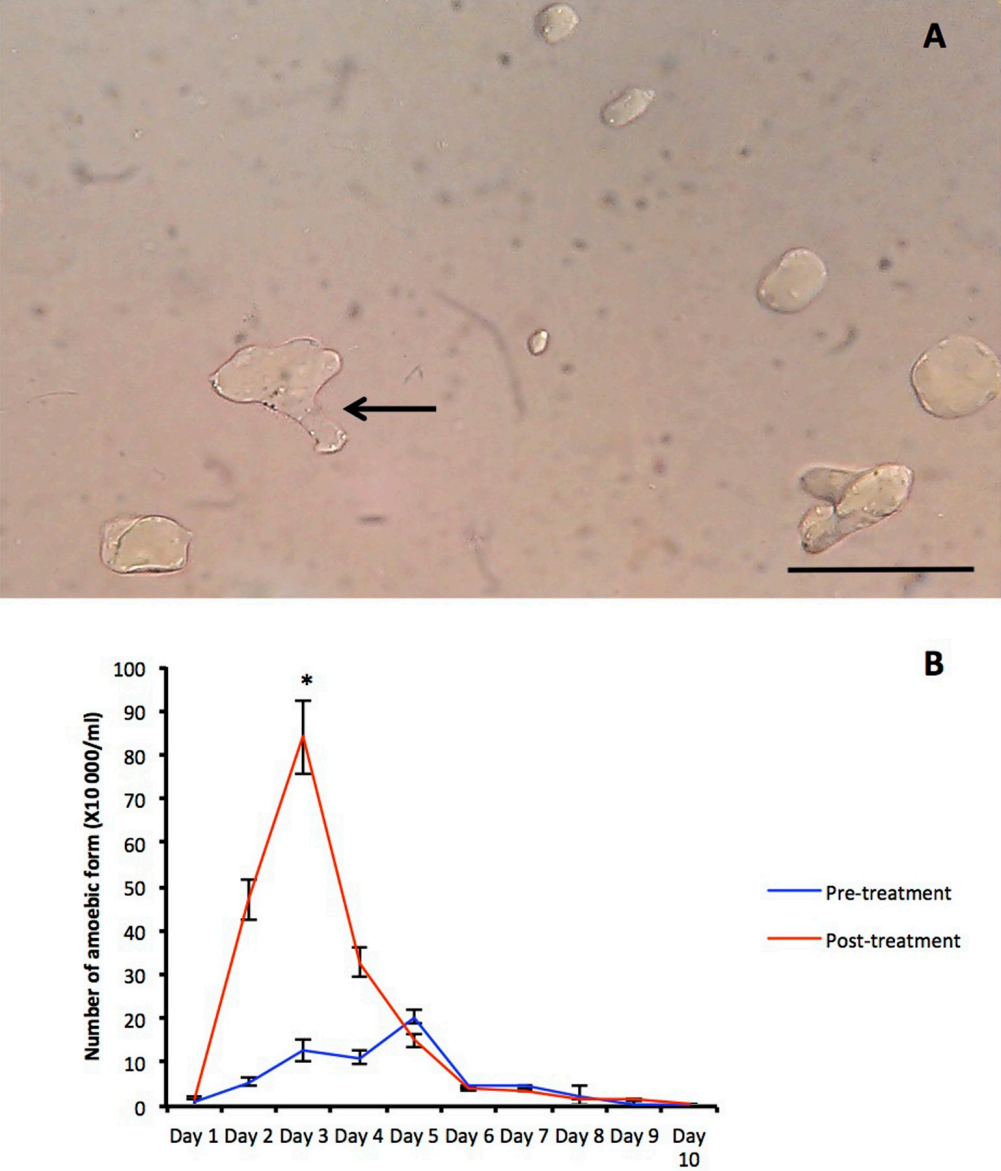
Amoebic forms seen in *Blastocystis* sp. isolated from urban symptomatic individuals. (A) Amoebic forms (arrow) seen under microscope at 40X magnification coexisting with vacuolar forms. Amoebic forms are seen with protrusion of the cytoplasm. Bar = 10μm (B) Increase in the number of amoebic morphology upon 0.001 mg/ml metronidazole treatment (Post-treatment) compared to the control (Pre-treatment). Amoebic forms were only observed in isolates from symptomatic urban individuals. Values are expressed as mean ± SD of 5 isolates. *represent P<0.001 in Students t-test comparing means of peak amoebic formation count in control and metronidazole treated isolates.

Amoebic forms of *Blastocystis* sp. were observed only in isolates obtained from symptomatic urban individuals. Treatment with metronidazole significantly increased number of amoebic cells in these isolates where the cells reached peak cell count on day 3 during treatment compared to the controls, which peaked on day 5. There was no formation of amoebic cells in treated and untreated cultures of *Blastocystis* sp. isolated from asymptomatic urban individuals and orang asli population (Fig 3).

**Fig 4:**
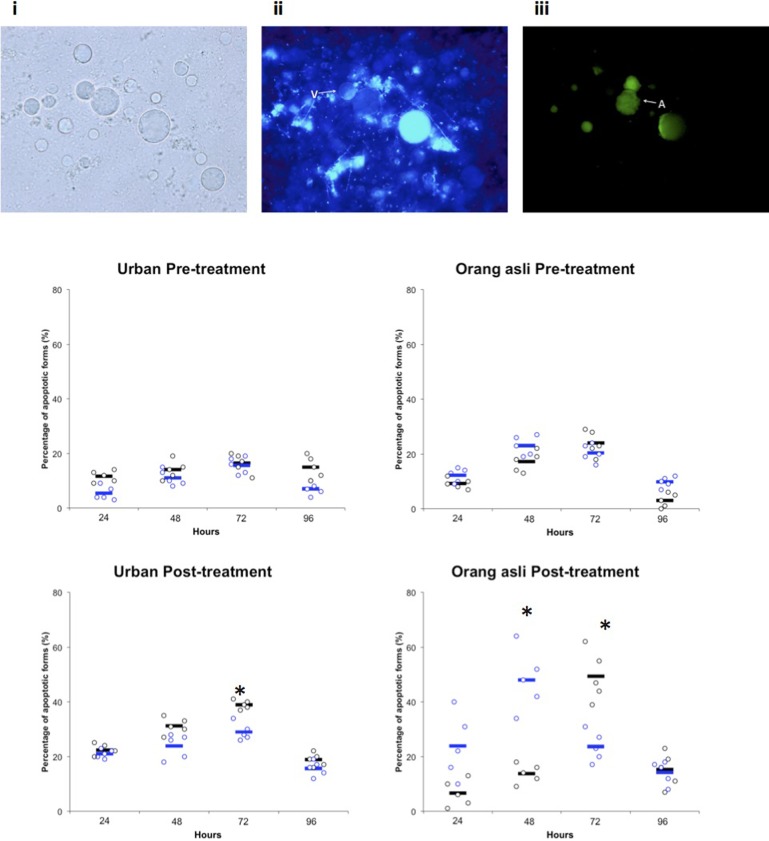
Staining of *Blastocystis* sp. cells to identify apoptosis and rate of apoptosis in control (Pre-treatment) and 0.001 mg/ml metronidazole treated (Post-treatment) *Blastocystis* sp. isolated from urban and orang asli individuals. (i) Light microscopy image of 0.001 mg/ml drug treated *Blastocystis* sp.(ii) Fluorescence image of parasite cells stained blue with Hoechst stain binding to DNA and staining nuclei in the periphery of the viable parasite(V). (iii) Fluorescence image of parasite stained with fluorescein isothiocyanate (FITC) labeled Annexin V identifying the apoptotic cells (A) through the binding with phosphatidylserine at the outer cell surface. The scatterplots show percentage of apoptotic forms in five *Blastocystis* sp. isolates. Black and blue circles represent symptomatic and asymptomatic isolates respectively. The black and blue lines represent the mean of respective data. Significantly higher rate of apoptosis was seen in isolates obtained from orang asli individuals. *represents P = 0.001 in Students t-test for comparison of percentage of apoptotic forms between pre- and post-treatment.

*Blastocystis* sp. cultures showed significant increase in the apoptotic forms when treated with metronidazole (Fig 4). There was significantly higher percentage of apoptotic forms in isolates obtained from orang asli individuals than urban persons (P = 0.045). In both urban and orang asli, isolates obtained from symptomatic individual harbored raised percentage of apoptotic forms than isolates obtained from asymptomatic individuals. There was a positive correlation between the apoptotic forms and number of viable cells found in the culture (r = 0.9712; P<0.05).

**Fig 5:**
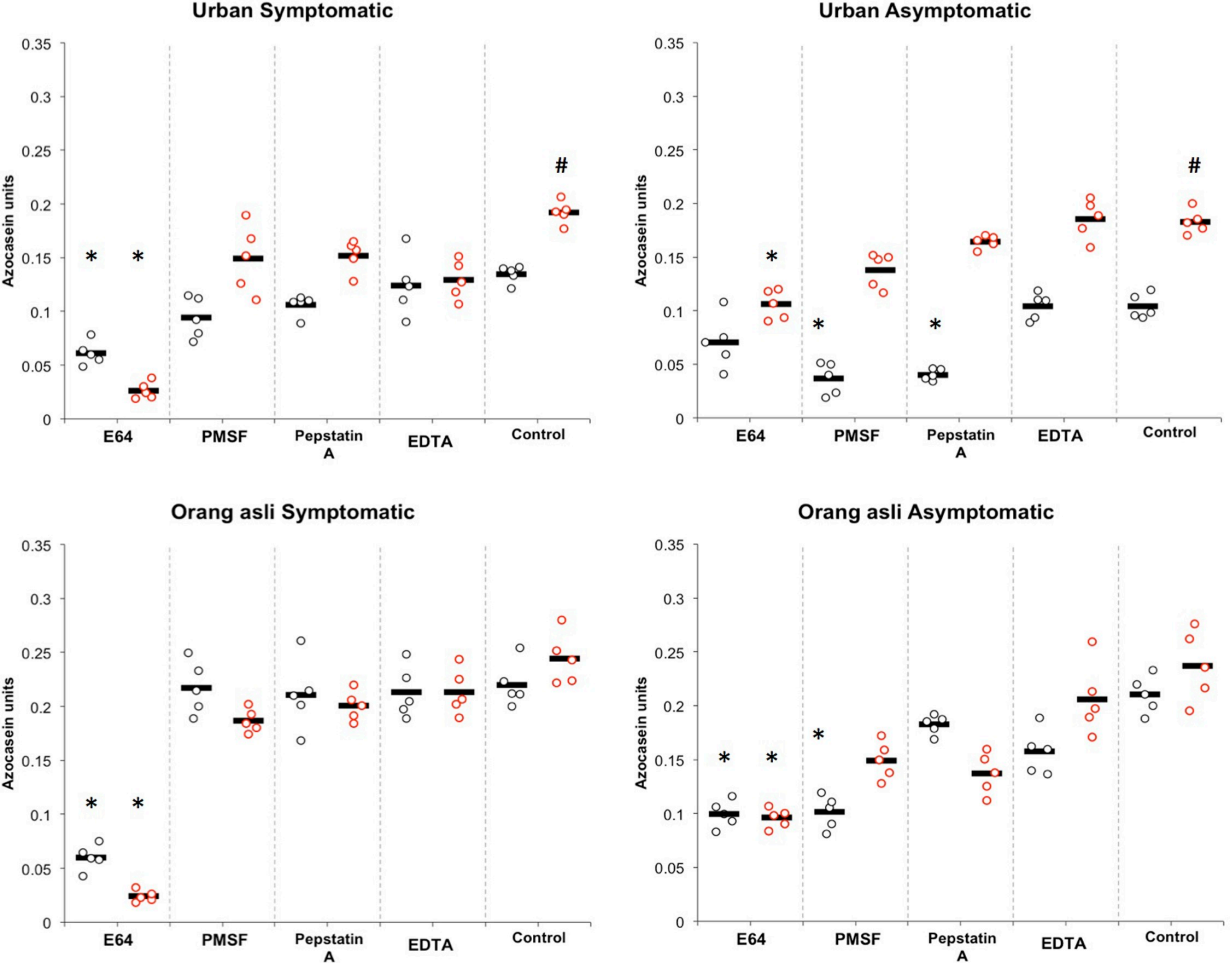
Specific protease activities in control (Pre-treatment) and 0.001 mg/ml metronidazole treated (Post-treatment) *Blastocystis* sp. isolated from urban and orang asli individual. Black circles and red circles represent protease activity of pre-treated and post-treated isolates respectively. The black line represent the mean(SD) of the 5 isolates tested. Note the predominance of cysteine protease upon treatment in *Blastocystis* sp. isolated from urban and orang asli individuals. *represents P<0.001 for the comparison with the mean of control. ^#^represents P<0.001 in Students t-test for comparison of protease activity pre- and post treatment.

Cysteine protease and serine protease were predominantly observed in *Blastocystis* sp. isolated from symptomatic and asymptomatic of urban and orang asli individuals. Upon metronidazole treatment, there was increase in protease activity in *Blastocystis* sp. isolated from both urban and orang asli individuals (Fig 5). A significant rise in proteases (P<0.05) was seen only in isolates obtained from urban individual where the predominant type of proteases, which were initially cysteine and serine in symptomatic and asymptomatic isolates respectively, reverted to predominant presence of cysteine type in both. Similar observation was seen in isolates obtained from orang asli individual where cysteine proteases were seen to be high in isolates obtained from symptomatic and asymptomatic individuals post-treatment. In short, isolates obtained from both urban and orang asli showed an increased level of protease activity in symptomatic as well as asymptomatic conditions with cysteine protease as the predominant type. However, significant effect of treatment was only observed in urban isolates.

**Fig 6:**
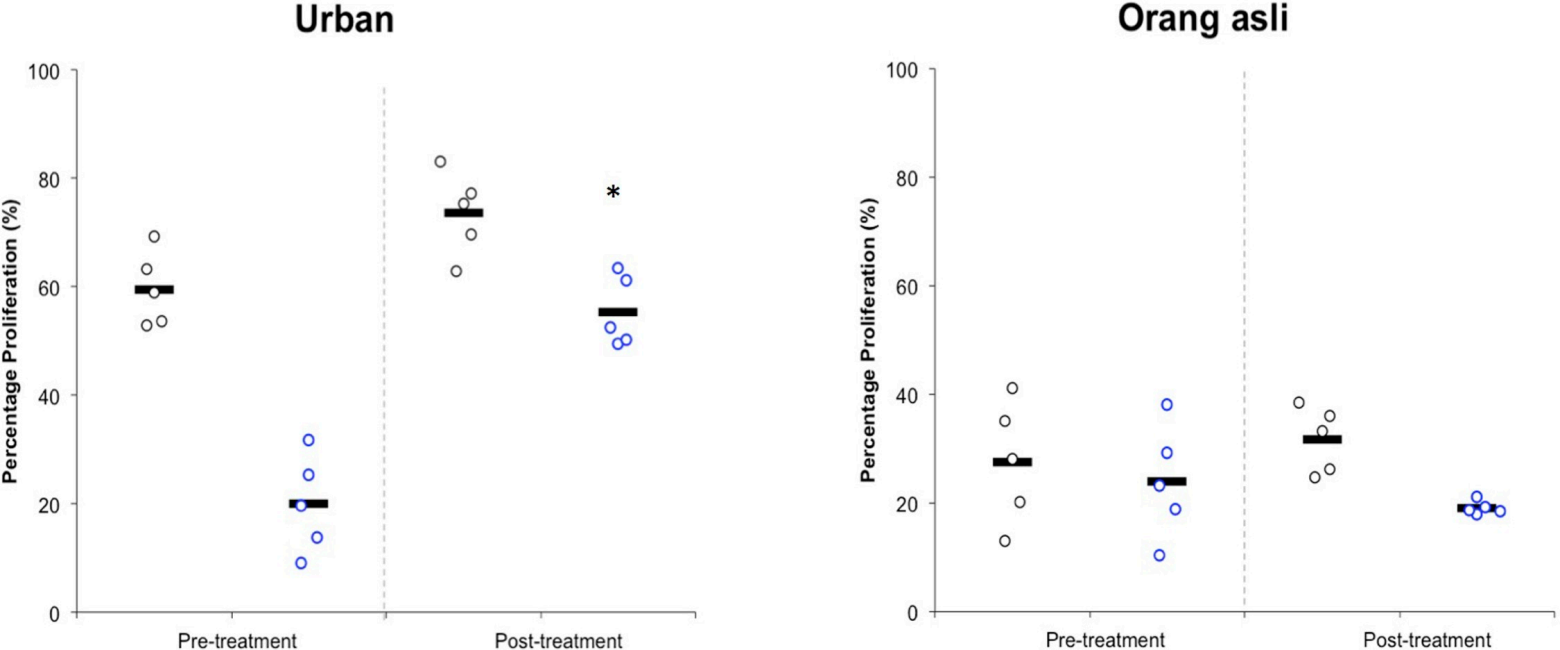
Effect of antigens from control (Pre-treatment) and 0.001 mg/ml metronidazole treated (Post-treatment) *Blastocystis* sp. on the proliferative ability of colon cancer cell, HCT116. The black and blue circles represent antigens extracted from symptomatic and asymptomatic isolates respectively. The black lines represent the mean of 5 isolates. Note the increased proliferative ability in antigens of *Blastocystis* sp. isolated from urban individuals upon metronidazole treatment. *represents P<0.001 in Students t-test for comparison of mean between pre- and post-treatment.

The current study also attempted to investigate the effect of metronidazole treated parasites on the proliferation of cancer calls *in vitro*. *Blastcystis* sp. isolated from urban individuals, upon treatment demonstrated an increase in the proliferation when triggered with antigens from both symptomatic and asymptomatic isolates. Metronidazole treated isolates from asymptomatic individuals exhibited increased proliferation in cancer cells. However, treatment on *Blastocystis* sp. from orang asli individuals did not show any significant difference to proliferation of cancer cells (Fig 6).

## Discussion

Metronidazole is the most commonly deployed drug for the treatment of *Blastocystis* sp. infection. Its prescription in adults involves dosages ranging from 250–800 mg, 3 times a day for 10 days or given in combination with other drugs such as paramomycin [25], co-trimoxazole [26] and trimethoprim-sulfamethoxazole [27] at varying dosages. In pediatric treatment, metronidazole is prescribed based on patient’s body weight at the dosage of 20–30 mg/kg/day [17].

Treatment failure of metronidazole have been reported as early as in 1976 [28]. Various other reports subsequently observed similar cases of treatment failure and resistance towards metronidazole. Despite being the first line of treatment for *Blastocystis* sp. infection, efficacy of metronidazole treatment ranges from anywhere between 0% to 100% with varying effective dosage from 250-750mg [29, 30]. Previous reports have demonstrated varying metronidazole resistance and evidence on mitochondrion-like structure when treated with metronidazole suggesting the need for energy [13]. There are currently studies carried out to investigate the efficacy of different drugs and its respective regimes on infected patients [3, 31] but there has been no study conclusively showing the effect of metronidazole on the pathogenicity of *Blastocystis* sp..

The present study reports on the pathogenic consequence in *Blastocystis* sp. due to inappropriate treatment administration. Previously, studies have reported that possible reason for failure in metronidazole treatment could be due to the absence of *nim* and *ntr* genes (genes responsible for the activation and inactivation of metronidazole into a toxic form) in the parasite[32]. Furthermore, inability of metronidazole to be at high concentration in the lumen suggest its failure in complete clearance of *Blastocystis* sp. [27]. In this study we observed at a concentration of 0.001 mg/ml, the parasite was not only resistant but triggered higher parasite growth. This provided a strong evidence for a possible mechanism that the parasite might undertake in order to evade the effect of metronidazole treatment at low concentrations. Other factors such as heightened chance of re-infection in endemic regions [33] and the influence of gut flora [30] may also result in treatment ineffectiveness.

Cysteine proteases have been reported to play an established role as virulent factor in many protozoan parasites such as *Entamoeba histolytica* and *Giardia* sp.[34] Proteases from *Blastocystis* sp. have not been clearly described and its association to pathogenicity has not gained conclusive evidence. Nevertheless, recent studies reporting on the predominance of cysteine protease in *Blastocystis* sp. [23] and its ability to degrade immunoglobulin A [35], increase in intestinal epithelial permeability by rearrangement of tight junction complex[36] and elicit IL-8 inflammatory response in *in vitro* condition[37] identifies the promising role of cysteine protease as a virulent factor of this parasite. Two secretory cysteine proteases from *Blastocystis* sp. have been identified through mass spectrometry [38]. These proteases were reported to be the virulent factor responsible for intestinal pathologies. Moreover, complete genome sequencing of *Blastocystis* sp. ST7 revealed 22 genes coding for proteases that was predicted to be molecular candidates for pathogenesis[39]. Out of these, 20 coded for cysteine protease, 1 for serine protease and 1 aspartic protease.

The present study concurs with previous findings[23] where we saw the predominance of cysteine protease in symptomatic isolates. For the first time, this study also reports the presence of serine protease in asymptomatic isolates of *Blastocystis* sp.. The exact role of serine protease seen in these isolates is unclear and more studies will be needed to characterize and understand its function. A physiological role of serine proteases in protozoan parasites such as *Entamoeba* sp. and *Acanthemoeba castellanii* has been reported [40, 41]. *Entamoeba invadens* possessed serine protease that was involved in excystation and metacystic development [40]. On the other hand, *Acanthemoeba castellanii* trophozoite and cysts treated separately with serine protease inhibitors demonstrated decreased number of cysts and tropozhoite formation respectively suggesting a pivotal role of serine protease in trophozoite differentiation and excystation [41]

In the present study, significant increase of cysteine protease levels in metronidazole treated *Blastocystis* sp. isolated from urban individuals imply that resistance to treatment can enhance the pathogenic potential of *Blastocystis* sp. However, isolates obtained from orang asli individuals showed insignificant change in protease activity upon treatment in comparison to urban isolates. We have witnessed in our laboratory, where *Blastocystis* sp. isolated from orang asli showed heightened robustness evidenced by the cysts not lysing in distilled water compared to isolates from urban individuals. The robustness seen in this *Blastocystis* sp. isolates could be the reason for insignificant response towards metronidazole. The response of isolates obtained from orang asli towards metronidazole treatment was however limited only to the apoptotic mechanism and increase in cell numbers. Therefore, more studies are warranted to characterize the *Blastocystis* sp. isolated from urban and orang asli individuals.

Studies in the past have suggested differential expression of proteases due to changing conditions of the protozoan parasite, *Entamoeba histolytica*. This parasite naturally expresses 86 genes for proteases coding for cysteine, serine, aspartic and metallo proteases and mostly with non-described functions. However, axenization altered the protease expression where some cysteine protease were highly expressed and some serine and metallo-proteases were expressed only intermediately[42]. In another similar study, *Entamoeba histolytica* exposed to heat stress were observed to express higher cysteine protease[43]. In contrast, another study have also reported that heat stress at 42°C causes 4–6 fold reduction in cysteine proteases but increased expression (up to 2-fold) of metalloproteases[42]. These studies suggest the influence of changing environmental conditions in the expression of proteases in parasitic protozoa. Similarly, in the present study, for the first time, expression of protease was demonstrated to change upon metronidazole treatment. Levels of cysteine protease increased and expression of predominant protease in asymptomatic isolates was seen to change from serine to cysteine type upon metronidazole treatment at 0.001 mg/ml. This data not only points to the possibility of *Blastocystis* sp. altering its protease expression in different conditions, but also suggest the possibility of dormant asymptomatic parasite reverting to active symptomatic ones. However, this postulation must be verified using molecular studies.

GI symptoms have been shown to persist in patients even after treatment with metronidazole [44]. Recently, we have reported 2 cases of patients experiencing exacerbation of gastrointestinal symptoms when treated with metronidazole[45]. The continued exacerbation and worsening of symptoms could be directly linked to increase in the cysteine protease levels in both *Blastocystis* sp. due to resistance towards metronidazole treatment. Whereas in asymptomatic *Blastocystis* sp. infection, if at all metronidazole treatment was administered and with inappropriate dosage, there are high possibilities of *Blastocystis* sp, reverting to symptomatic forms.

Presence of amoebic forms had been associated to pathogenicity in *Blastocystis* sp. Studies carried on previously reported high occurrence of amoebic forms in symptomatic isolates over asymptomatic isolates [5, 46]. The ultrastructural studies on amoebic forms of *Blastocystis* sp. exhibited the presence of surface coat, which facilitates adherence to the intestinal walls and surrounding bacteria[47]. The surface of amoebic form has also been shown to possess strong fluorescent lectin binding sites suggesting surface properties that may imply pathogenic potential[48]. Another study reported on transition of vacuolar and granular forms to amoebic forms in patients who showed progression from asymptomatic to symptomatic state[49]. Our previous finding showed a positive correlation of amoebic forms in culture with protease activity[22]. The accumulated evidence further strengthens the pathogenic role of amoebic forms. In this study, we saw an increase in the amoebic forms in *Blastocystis* sp. isolated from symptomatic urban individuals upon metronidazole treatment. This evidence could further suggest increased pathogenicity due to treatment at inappropriate concentration of metronidazole.

Parasites isolated from orang asli individuals demonstrated an increase in parasite numbers and this can be due to higher apoptotic formation when treated with metronidazole. Apoptosis in *Blastocystis* sp. was shown previously to be subtype related as higher rate of apoptosis in ST 3 against other subtypes was reported (Dhurga et al. 2012). In the present study, *Blastocystis* sp. ST 3 not only showed apoptosis but its rates differed between isolates obtained from urban and orang asli suggesting that apoptosis could be influenced by the community of locality where the parasite is isolated from. Previous *in vitro* study reported when *Blastocystis* sp. was isolated from Malaysian, Indonesian, Singaporean and Pakistan population the parasite showed difference in the resistance towards metronidazole [14]. This concurred with our study where difference in metronidazole resistance was seen among different subtypes tested [50]. This suggests that response to metronidazole is highly variable.

The current study showed strong evidence of varying response towards metronidazole in *Blastocystis* sp. isolated from individuals within the Malaysian population (Urban and orang asli). Orang asli population and settlements, regarded to be the most rural in Malaysia, live on high fibre diet comprising of natural foods and mostly depend on agricultural economy for living (based on personal observation during sample collection). These type of populations are reported to have an increased microbial and functional diversity of the gut flora[15, 51]. In contrary to this, the urban population lives a lifestyle consuming highly industrial and processed food rich in simple sugars and fats. As a result this population reportedly, have lost a great deal of diversity especially that of the beneficial and protective bacteria[52]. *Blastocystis* sp. isolated from these environments may possess distinct phenotypic characteristics that could have resulted the variation in response towards metronidazole. Therefore, population-based variation of resistance towards metronidazole discussed above could also be explained by the distinction of gut environment in the studied populations.

Previously, a study reported the ability of solubilized antigen from symptomatic isolates induced greater percentage of cancer cell proliferation compared to asymptomatic isolates [53]. Subsequently, another study reported that symptomatic ST 3 could trigger greater proliferation of cancer cells with immune alterations[7]. These data suggest that the ability to proliferate cancer cells by *Blastocystis* sp. solubilized antigens can be well used to distinguish alterations of pathogenic potential. In this study, drug treated urban symptomatic and asymptomatic isolates stimulated significantly higher cell proliferation in cancer cells. We also have observed that the raise in proliferation of cancer cells coincided with increasing cysteine protease activity of *Blastocystis* sp. This suggests the possible role of cysteine protease in inducing cell proliferation. However, more study is needed to confirm this postulation. Previous report suggested screening for *Blastocystis* sp. in colorectal cancer patients as it could facilitate the tumor growth[21]. Based on results from the current study, deploying metronidazole treatment in cancer patients with *Blastocystis* sp. infection should be reconsidered as this shows the possibility of increasing the pathogenic potentials of the organism resulting in exacerbated symptoms in immune-compromised cancer patients.

**Fig 7:**
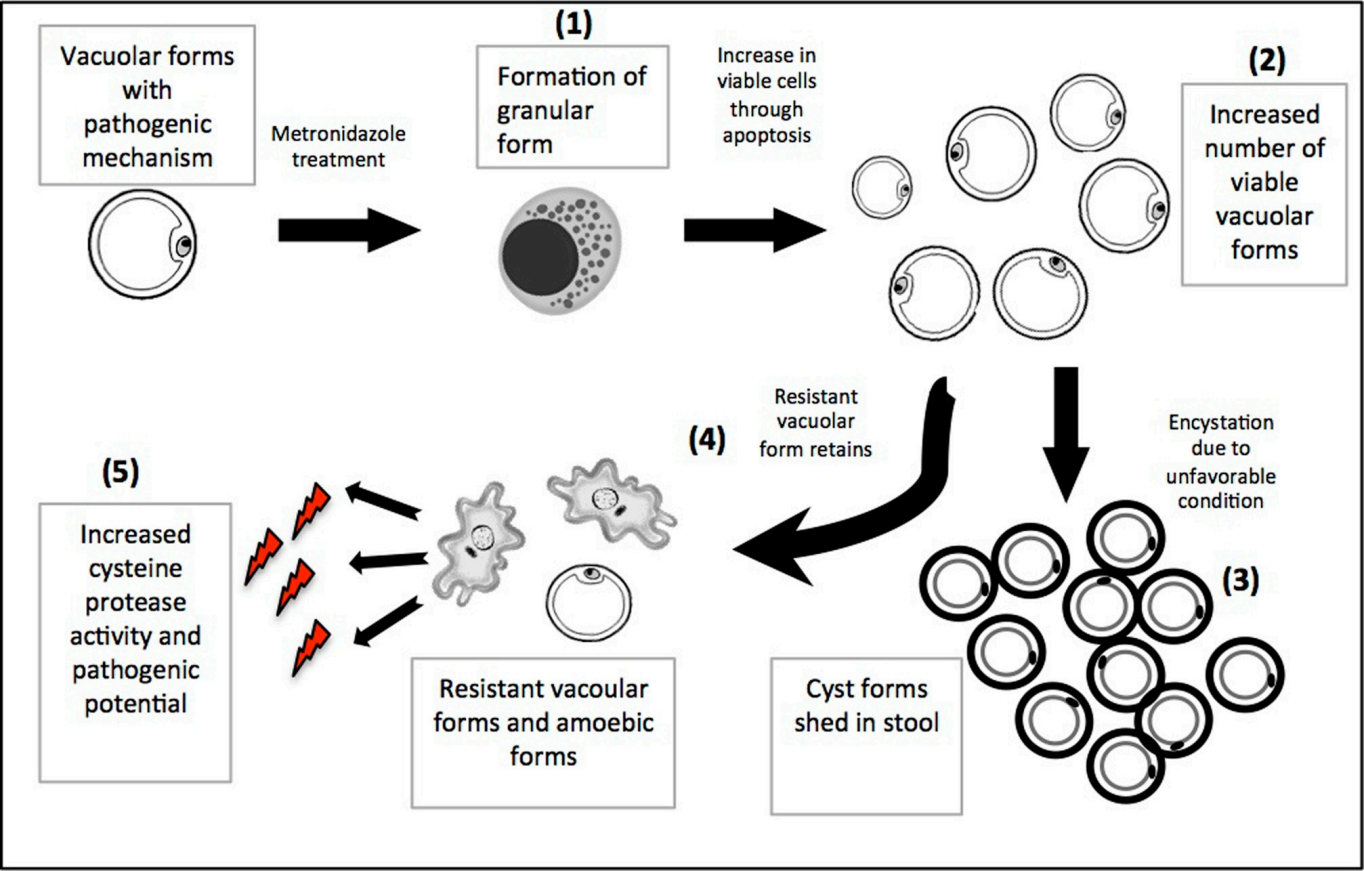
Schematic diagram depicting the major findings in the current study. Metronidazole treatment at a certain concentration (0.001 mg/ml) heightens the parasite number. **(1), (2)** The parasite cells forms high number if granular forms that in turn produce viable vacuolar forms. **(3)** When the condition turn unfavourable, the parasites may encyst and excreted in large numbers as reported in a case study previously. **(4)** It could be that some vacuolar forms that is metronidazole resistant *Blastocystis* sp. would retain. **(5)** This resistant vacoular forms and increased amoebic forms of *Blastocystis* sp. exhibit increased pathogenic potentials (as shown by the thunder symbols) in terms of greater cysteine protease activity, amoebic forms and ability to proliferate cancer cells.

Solubilized antigen was extracted from *in vitro* culture of *Blastocystis* sp., which was purified to minimal bacterial contamination. This condition, compared to the axenic ones mimics the natural environment of *Blastocystis* sp. and better explains its mechanisms. However, a control experiment with only bacterial solubilized antigens showed negligible protease activity and proliferative ability of cancer cells. In conclusion, this study provided evidence for possible pathophysiological effect of metronidazole treatment on *Blastocystis* sp., which varies depending on the source where the parasite is isolated from. *Blastocystis* sp. exposed to a specific concentration of metronidazole does develop resistance, which in turn increase its cell numbers and gain more pathogenic potentials such as protease activity, amoebic formations and ability in cancer cell proliferation. A sschematic diagram depicting the major findings in the current study is provided (Fig 7). However, more in depth functional studies on cysteine proteases as well as cellular and molecular mechanisms in metronidazole treated *Blastocystis* sp. would shed light in understanding the resistance towards the drug and pathogenicity. It is essential to elucidate the role of gut flora on *Blastocystis* sp. treatment modalities. Re-assessment of treatment options on *Blastocystis* sp. should be carried out to find better for drug with greater efficacy.

## Supporting information

S1 Questionnaire Questionnaire used for the sample collection. The questionnaire was used to obtain relevant details.
(PDF)

## References

[1] Scanlan PD. *Blastocystis*: past pitfalls and future perspectives. Trends in Parasitology. 2012;28(8):327–34. 10.1016/j.pt.2012.05.001 22738855

[2] Graczyk TK, Shiff CK, Tamang L, Munsaka F, Beitin AM, Moss WJ. The association of Blastocystis hominis and Endolimax nana with diarrheal stools in Zambian school-age children. Parasitology research. 2005;98(1):38–43. 10.1007/s00436-005-0003-0 16249910

[3] Roberts T, Stark D, Harkness J, Ellis J. Update on the pathogenic potential and treatment options for *Blastocystis* sp. Gut Pathogens. 2014;6:17 10.1186/1757-4749-6-17 24883113 PMC4039988

[4] Deeb HKE, Salah-Eldin H, Khodeer S. *Blastocystis hominis* as a contributing risk factor for development of iron deficiency anemia in pregnant women. Parasitology Research. 2012;110(6):2167–74. 10.1007/s00436-011-2743-3 22167376

[5] Katsarou-Katsari A, Vassalos CM, Tzanetou K, Spanakos G, Papadopoulou C, Vakalis N. Acute Urticaria Associated with Amoeboid Forms of Blastocystis sp. Subtype 3. Acta Dermato-Venereologica. 2008;88(1):80–1. 10.2340/00015555-0338 18176765

[6] Khademvatan S, Masjedizadeh R, Rahim F, Mahbodfar H, Salehi R, Yousefi-Razin E, et al *Blastocystis* and irritable bowel syndrome: Frequency and subtypes from Iranian patients. Parasitology international. 2017;66(2):142–5. 10.1016/j.parint.2017.01.005 28087441

[7] Kumarasamy V, Kuppusamy UR, Samudi C, Kumar S. Blastocystis sp. subtype 3 triggers higher proliferation of human colorectal cancer cells, HCT116. Parasitolgy Research. 2013;112(10):3551–5. 10.1007/s00436-013-3538-5 23933809

[8] Stensvold CR, Christiansen DB, Olsen KEP, Nielsen HV. Short Report: *Blastocystis* sp. Subtype 4 is Common in Danish *Blastocystis*-Positive Patients Presenting with Acute Diarrhea. American Journal of Tropical Medicine and Hygiene. 2011;84(6):883–5. 10.4269/ajtmh.2011.11-0005 21633023 PMC3110361

[9] Vogelberg C, Stensvold CR, Monecke S, Ditzen A, Stopsack K, Heinrich-Gräfe U, et al *Blastocystis* sp. subtype 2 detection during recurrence of gastrointestinal and urticarial symptoms. Parasitology international. 2010;59(3):469–71. 10.1016/j.parint.2010.03.009 20363362

[10] Scanlan PD, Stensvold CR, Rajilić‐Stojanović M, Heilig HGHJ, De Vos WM, O'Toole PW, et al The microbial eukaryote *Blastocystis* is a prevalent and diverse member of the healthy human gut microbiota. FEMS microbiology ecology. 2014;90(1):326–30. 10.1111/1574-6941.12396 25077936

[11] Mitanshu S, Bryan TC, Dhyan R, Shadab A, Krishnaiyer S, Kaleem R, et al *Blastocystis hominis* and *Endolimax nana* Co-Infection Resulting in Chronic Diarrhea in an Immunocompetent Male. Case reports in gastroenterology. 2012;6(2):358–64. 10.1159/000339205 22740811 PMC3383306

[12] Wu Z, Mirza H, Tan KSW. Intra-Subtype Variation in Enteroadhesion Accounts for Differences in Epithelial Barrier Disruption and Is Associated with Metronidazole Resistance in Blastocystis Subtype-7. PLOS Neglected Tropical Disease. 2014;8(5):e2885. 10.1371/journal.pntd.0002885 PMC4031124 24851944

[13] Raman K, Kumar S, Chye TT. Increased number of mitochondrion-like organelle in symptomatic *Blastocystis* subtype 3 due to metronidazole treatment. Parasitology research. 2016;115(1):391–6. 10.1007/s00436-015-4760-0 26481491

[14] Haresh K, Suresh K, Anuar AK, Saminathan S. Isolate resistance of *Blastocystis hominis* to metronidazole. Tropical Medicine & International Health. 1999;4(4):274–7. 10.1046/j.1365-3156.1999.00398.x 10357863

[15] Tyakht AV, Alexeev DG, Popenko AS, Kostryukova ES, Govorun VM. Rural and urban microbiota: to be or not to be? Gut Microbes. 2014;5(3):351–6. 10.4161/gmic.28685 24691073 PMC4153773

[16] De Filippo C, Cavalieri D, Di Paola M, Ramazzotti M, Poullet JB, Massart S, et al Impact of diet in shaping gut microbiota revealed by a comparative study in children from Europe and rural Africa. Proceedings of the National Academy of Sciences. 2010;107(33):14691–6. 10.1073/pnas.1005963107 PMC2930426 20679230

[17] Kurt Ö, Al FD, Tanyüksel M. Eradication of *Blastocystis* in humans: Really necessary for all? Parasitology international. 2016;65(6):797–801. 26780545 10.1016/j.parint.2016.01.010

[18] Leelayoova S, Taamasri P, Rangsin R, Naaglor T, Thathaisong U, Mungthin M. In-vitro cultivation: a sensitive method for detecting *Blastocystis hominis*. Annals of tropical medicine and parasitology. 2002;96(8):803–7. 10.1179/000349802125002275 12625935

[19] Ragavan ND, Govind SK, Chye TT, Mahadeva S. Phenotypic variation in Blastocystis sp. ST3. Parasites & vectors. 2014;7(1):404. 25174569 10.1186/1756-3305-7-404 PMC4261759

[20] Dhurga D, Suresh K, Tan T, Chandramathi S. Apoptosis in *Blastocystis* spp. is related to subtype. Transactions of the Royal Society of Tropical Medicine and Hygiene. 2012;106(12):725–30. 10.1016/j.trstmh.2012.08.005 23141370

[21] Chandramathi S, Suresh K, Kuppusamy UR. Solubilized antigen of *Blastocystis hominis* facilitates the growth of human colorectal cancer cells, HCT116. Parasitology research. 2010;106(4):941–5. 10.1007/s00436-010-1764-7 20165878

[22] Rajamanikam A, Govind SK. Amoebic forms of Blastocystis spp.—evidence for a pathogenic role. Parasites & Vectors. 2013;6:295. 24499467 10.1186/1756-3305-6-295 PMC3853151

[23] Sio SWS, Puthia MK, Lee ASY, Lu J, Tan KSW. Protease activity of *Blastocystis hominis*. Parasitology Research. 2006;99(2):126–30. 10.1007/s00436-006-0131-1 16518611

[24] Khan NA, Jarroll EL, Panjwani N, Cao Z, Paget TA. Proteases as Markers for Differentiation of Pathogenic and Nonpathogenic Species of *Acanthamoeba*. Journal of Clinical Microbiology. 2000;38(8):2858–61. 10921939 10.1128/jcm.38.8.2858-2861.2000 PMC87129

[25] Pasqui AL, Savini E, Saletti M, Guzzo C, Puccetti L, Auteri A. Chronic urticaria and Blastocystis hominis infection. A case report. European review for medical and pharmacological sciences. 2004;8:117–20. 15368795

[26] Andiran N, Acikgoz ZC, Turkay S, Andiran F. *Blastocystis hominis*—an emerging and imitating cause of acute abdomen in children. Journal of pediatric surgery. 2006;41(8):1489–91. 10.1016/j.jpedsurg.2006.04.037 16863863

[27] Stensvold Arendrup MC, Nielsen HV, Bada A, Thorsen S. Symptomatic infection with *Blastocystis* sp. subtype 8 successfully treated with trimethoprim–sulfamethoxazole. Annals of tropical medicine and parasitology. 2008;102(3):271–4. 10.1179/136485908X278847 18348782

[28] Zierdt CH, Tan H. Ultrastructure and light microscope appearance of Blastocystis hominis in a patient with enteric disease. Zeitschrift fur parasitenkunde-parasitology research. 1976;50(3):277–83. 10.1007/BF02462972 997721

[29] Coyle CM, Varughese J, Weiss LM, Tanowitz HB. *Blastocystis*: to treat or not to treat…. Clinical infectious diseases. 2011;54(1):105–10. 10.1093/cid/cir810 22075794

[30] Stensvold CR, Smith HV, Nagel R, Olsen KE, Traub RJ. Eradication of *Blastocystis* carriage with antimicrobials: reality or delusion? Journal of clinical gastroenterology. 2010;44(2):85–90. 10.1097/MCG.0b013e3181bb86ba 19834337

[31] Sekar U, Shanthi M. Blastocystis: Consensus of treatment and controversies. Tropical parasitology. 2013;3(1):35 10.4103/2229-5070.113901 23961439 PMC3745668

[32] Pal D, Banerjee S, Cui J, Schwartz A, Ghosh SK, Samuelson J. Giardia, Entamoeba, and Trichomonas enzymes activate metronidazole (nitroreductases) and inactivate metronidazole (nitroimidazole reductases). Antimicrobial agents and chemotherapy. 2009;53(2):458–64. 10.1128/AAC.00909-08 19015349 PMC2630645

[33] Nigro L, Larocca L, Massarelli L, Patamia I, Minniti S, Palermo F, et al A Placebo‐Controlled Treatment Trial of *Blastocystis hominis* Infection with Metronidazole. Journal of travel medicine. 2003;10(2):128–30. 12650658 10.2310/7060.2003.31714

[34] McKerrow JH, Sun E, Rosenthal PJ, Bouvier J. The Proteases and Pathogenicity of Parasitic Protozoa. Annual Review of Microbiology. 1993;47: 821–53. 10.1146/annurev.mi.47.100193.004133 8257117

[35] Puthia MK, Vaithilingam A, Lu J, Tan KSW. Degradation of human secretory immunoglobulin A by *Blastocystis*. Parasitology Research. 2005;97(5):386–9. 10.1007/s00436-005-1461-0 16151742

[36] Mirza H, Wu Z, Teo JDW, Tan KSW. Statin pleiotropy prevents rho kinase-mediated intestinal epithelial barrier compromise induced by *Blastocystis* cysteine proteases. Cellular Microbiology. 2012;14(9):1474–84. 10.1111/j.1462-5822.2012.01814.x 22587300

[37] Puthia MK, Lu J, Tan KSW. *Blastocystis ratti* Contains Cysteine Proteases That Mediate Interleukin-8 Response from Human Intestinal Epithelial Cells in an NF-kB-Dependent Manner. Eukaryotic Cell. 2008;7(3):435–43. 10.1128/EC.00371-07 18156286 PMC2268520

[38] Wawrzyniak I, Texier C, Poirier P, Viscogliosi E, Tan KSW, Delbac Fdr, et al Characterization of two cysteine proteases secreted by *Blastocystis* ST7, a human intestinal parasite. Parasitology International. 2012;61(3):437–42. 10.1016/j.parint.2012.02.007 22402106

[39] Denoeud F, Roussel Ml, Noel B, Wawrzyniak I, Silva CD, Diogon M, et al Genome sequence of the stramenopile *Blastocystis*, a human anaerobic parasite. Genome Biology. 2011;12(3):R29 10.1186/gb-2011-12-3-r29 21439036 PMC3129679

[40] Makioka A, Kumagai M, Kobayashi S, Takeuchi T. Involvement of serine proteases in the excystation and metacystic development of *Entamoeba invadens*. Parasitology research. 2009;105(4):977 10.1007/s00436-009-1478-x 19479279

[41] Dudley R, Alsam S, Khan NA. The role of proteases in the differentiation of Acanthamoeba castellanii. FEMS microbiology letters. 2008;286(1):9–15. 10.1111/j.1574-6968.2008.01249.x 18616591

[42] Tillack M, Biller L, Irmer H, Freitas M, Gomes MA, Tannich E, et al The *Entamoeba histolytica* genome: primary structure and expression of proteolytic enzymes. BMC genomics. 2007;8(1):170. 17567921 10.1186/1471-2164-8-170 PMC1913524

[43] Weber C, Guigon G, Bouchier C, Frangeul L, Moreira S, Sismeiro O, et al Stress by heat shock induces massive down regulation of genes and allows differential allelic expression of the Gal/GalNAc lectin in Entamoeba histolytica. Eukaryotic Cell. 2006;5(5):871–5. 10.1128/EC.5.5.871-875.2006 16682464 PMC1459685

[44] Roberts T, Ellis J, Harkness J, Marriott D, Stark D. Treatment failure in patients with chronic Blastocystis infection. Journal of medical microbiology. 2014;63(2):252–7. 24243286 10.1099/jmm.0.065508-0

[45] Rajamanikam A, Kumar S, Samudi C, Kudva M. Exacerbated symptoms in *Blastocystis* sp.-infected patients treated with metronidazole: two case studies. Parasitology research. 2018 10.1007/s00436-018-5948-x. 29872961

[46] Chye TT, Suresh KG. Predominance of amoeboid forms of *Blastocystis hominis* in isolates from symptomatic patients. Parasitology Research. 2006;98(3):189–93. 10.1007/s00436-005-0033-7 16323025

[47] Chye TT, Suresh KG. Amoeboid form of *Blastocystis hominis*—a detailed ultrastructural insight. Parasitology Research. 2006;99(6):737–42. 10.1007/s00436-006-0214-z 16816959

[48] Chye TT, Suresh KG, Smith HV. Phenotypic and genotypic characterisation of *Blastocystis hominis* isolates implicates subtype 3 as a subtype with pathogenic potential. Parasitology Research. 2008;104(1):85–93. 10.1007/s00436-008-1163-5 18795333

[49] Vassalos CM, Spanakos G, Vassalou E, Papadopoulou C, Vakalis N. Differences in Clinical Significance and Morphologic Features of *Blastocystis* sp Subtype 3. American Journal of Clinical Pathology. 2010;133(2):251–8. 10.1309/AJCPDOWQSL6E8DMN 20093234

[50] Girish S, Kumar S, Aminudin N. Tongkat Ali (Eurycoma longifolia): a possible therapeutic candidate against Blastocystis sp. Parasites & vectors. 2015;8(1):332. 26082155 10.1186/s13071-015-0942-y PMC4476169

[51] Moschen AR, Wieser V, Tilg H. Dietary factors: major regulators of the gut's microbiota. Gut and liver. 2012;6(4):411 10.5009/gnl.2012.6.4.411 23170142 PMC3493718

[52] Segata N. Gut microbiome: westernization and the disappearance of intestinal diversity. Current Biology. 2015;25(14):R611–R3. 10.1016/j.cub.2015.05.040 26196489

[53] Chan KH, Chandramathi S, Suresh K, Chua KH, Kuppusamy UR. Effects of symptomatic and asymptomatic isolates of *Blastocystis hominis* on colorectal cancer cell line, HCT116. Parasitology research. 2012;110(6):2475–80. 10.1007/s00436-011-2788-3 22278727

