# Supplementary material for: Resistance towards metronidazole in Blastocystis sp.: A pathogenic consequence
Source: PLoS One. 2019 Feb 22;14(2):e0212542. doi: 10.1371/journal.pone.0212542 (PMC6386359; doi:10.1371/journal.pone.0212542)
Supplement: S1 Questionnaire — The questionnaire was used to obtain relevant details. (PDF) [file pone.0212542.s001.pdf]

DEPARTMENT OF PARASITOLOGY

University of Malaya

Blastocystis Research Team

PATIENT'S DETAIL **NO: ________**

Name: ____________________________

Date of Birth: ____________________________

Age: ____________________________

Gender: ____________________________

Race: ____________________________

Occupation: ____________________________
(if animal handler, specify type of animals handled)

Address: ____________________________

(A) PATIENT'S CLINICAL HISTORY & TREATMENT

Sample obtained:

- Biopsy sample ( )
- Stool sample ( )
- Washout fluid sample ( )

Treatment(s) undergone before sample collection:

- Chemotherapy ( )
- Radiotherapy ( )
- Surgery ( )
- Recent antibiotic used (<1 month) ( )
- Recent antiprotozoal used (<1 month) Name & Dose:…………………………….( )

Tumor, if present, location in the bowel:

- Colon ( )
- Rectal ( )
- Others …………………..

Type of tumor:

- Benign ( )
- Malignant ( )
- Constitutional ( )
- Metastatic ( )

Symptoms and signs

- Frequent diarrhea ( )
- Difficulty in defecation ( )
- Any skin rash; *Please specify* ………………………………………….. ( )
- Blood mixed in the stools ( )
- Frequent flatulence ( )

Frequency of symptoms: ___________________ /week

Lifestyle

- Active, healthy ( )
- Active, busy ( )
- Passive ( )

Supplementations (example: vitamins) ( )

Any illness(s)

- Cardiovascular disease ( )
- Pulmonary disease ( )
- Diabetes ( )
- Others ………………….

Family history of colon cancer

- Anyone ( )
- Close relative before the age of 55 or multiple relatives ( )

How stressed were you at home?

**Least**					**Very**
1	2	3	4	5	6

How stressed were you in your workplace?

**Least**					**Very**
1	2	3	4	5	6

How happy were you in the past 1 month?

**Least**					**Very**
1	2	3	4	5	6

Any critical stress incidence(s) in the past 12 months

Job stress

- Being unhappy in your job ( )
- Pressure at work place ( )
- Very long working hours ( )
- Working under dangerous conditions ( )
- Being insecure about your chance for advancement or risk of termination ( )
- Having to give speeches in front of colleagues ( )
- Facing discrimination or harassment at work ( )

Life stress

- Death of loved ones ( )
- Accident ( )
- Any other traumatizing event (eg. Theft, bully, rape attempt) ( ) ; *Please specify*

  …………………………………………………………………………………………….

- Increase in financial obligation ( )
- Changed jobs ( )
- Family problems ( )
- Taking care of an elderly or sick family member ( )
- Emotional problems (depression, anxiety, anger, grief, guilt, low self-esteem) ( )
- Others; *Please specify*…………………………………………………………… ( )

Smoker ( )
If yes, how many sticks per day? ………..

Alcohol consumption ( )
If yes, how often? ……….. days in a week

Diets high in:
- Red meats ( )
- Vegetables ( )
- Fresh fruits ( )
- Poultry & fish ( )

END OF QUESTIONNAIRE
